# Development of a versatile HPLC-based method to evaluate the activation status of small GTPases

**DOI:** 10.1016/j.jbc.2021.101428

**Published:** 2021-11-19

**Authors:** Makoto Araki, Kaho Yoshimoto, Meguri Ohta, Toshiaki Katada, Kenji Kontani

**Affiliations:** 1 Department of Biochemistry, Meiji Pharmaceutical University, Tokyo, Japan; 2 Molecular Cell Biology Laboratory, Research Institute of Pharmaceutical Sciences, Faculty of Pharmacy, Musashino University, Tokyo, Japan

**Keywords:** high-performance liquid chromatography, small GTPase, Ras protein, RHEB, mammalian target of rapamycin, tuberous sclerosis complex, GTPase-activating protein, signal transduction, BSA, bovine serum albumin, dox, doxycycline, GAP, GTPase-activating proteins, GDI, guanine-nucleotide dissociation inhibitor, IP-RP-HPLC, ion-pair reversed-phase HPLC, mTORC1, mechanistic target of rapamycin complex 1, RHEB, Ras homolog enriched in brain, S6K, S6 kinase, TBA-B, tetrabutylammonium bromide

## Abstract

Small GTPases cycle between an inactive GDP-bound and an active GTP-bound state to control various cellular events, such as cell proliferation, cytoskeleton organization, and membrane trafficking. Clarifying the guanine nucleotide-bound states of small GTPases is vital for understanding the regulation of small GTPase functions and the subsequent cellular responses. Although several methods have been developed to analyze small GTPase activities, our knowledge of the activities for many small GTPases is limited, partly because of the lack of versatile methods to estimate small GTPase activity without unique probes and specialized equipment. In the present study, we developed a versatile and straightforward HPLC-based assay to analyze the activation status of small GTPases by directly quantifying the amounts of guanine nucleotides bound to them. This assay was validated by analyzing the RAS-subfamily GTPases, including HRAS, which showed that the ratios of GTP-bound forms were comparable with those obtained in previous studies. Furthermore, we applied this assay to the investigation of psychiatric disorder-associated mutations of RHEB (RHEB/P37L and RHEB/S68P), revealing that both mutations cause an increase in the ratio of the GTP-bound form in cells. Mechanistically, loss of sensitivity to TSC2 (a GTPase-activating protein for RHEB) for RHEB/P37L, as well as both decreased sensitivity to TSC2 and accelerated guanine-nucleotide exchange for RHEB/S68P, is involved in the increase of their GTP-bound forms, respectively. In summary, the HPLC-based assay developed in this study provides a valuable tool for analyzing small GTPases for which the activities and regulatory mechanisms are less well understood.

Small GTPases function as molecular switches through conformational changes between an inactive GDP-bound form and an active GTP-bound form. More than 150 small GTPases that are known in humans are classified into five distinct subfamilies (*i.e.*, RAS, RHO/RAC, RAB, ARF/ARL, and RAN) based on the similarities of their primary structure (1, 2). Small GTPases generally interact with effector proteins in their activated form (GTP-bound form) to regulate various biological events, such as cell proliferation and differentiation, cell motility, and intracellular transport. The balance between the activated and inactive forms of small GTPases is tightly regulated by several regulatory factors: guanine-nucleotide exchange factors promote the dissociation of GDP from small GTPases, thereby facilitating the binding of GTP (which is about 10 times more abundant than GDP in cells) to small GTPases. In turn, GTPase-activating proteins (GAPs) promote the conversion of GTP-bound forms to GDP-bound forms by stimulating the intrinsic GTPase activity of small GTPases. For the RHO and RAB proteins, a guanine-nucleotide dissociation inhibitor (GDI) protein (RHO-GDI and RAB-GDI, respectively) prevents the dissociation of GDP from small GTPases (3, 4, 5, 6).

Mutations in small GTPases that perturb the balance of their guanine-nucleotide binding states are associated with various diseases, including cancer. For example, the glycine 12 (G12) mutation in RAS, which is frequently observed in cancer, leads to RAS activation by preventing GAP-mediated GTP hydrolysis (7). The mutations in RAC1 (*e.g.*, P29S and N92I) identified in melanoma increase the proportion of activated RAC1 due to the enhanced GDP/GTP-exchange reactions associated with accelerated GDP dissociation (8, 9, 10, 11, 12, 13). Unlike these activating mutations, the ARL6/T31R mutation identified in ciliopathies causes reduced binding affinity for GTP, resulting in a constitutive GDP-bound (inactive) ARL6 (14, 15, 16). The clarification of the small GTPase activities and the impact of disease states on their activity are necessary for understanding the mechanisms of the physiological responses and drug-discovery targeting small GTPases. To date, several assays have been developed to analyze the activities of small GTPases; however, many small GTPases remain poorly understood in terms of the regulation of their guanine-nucleotide binding states in cells.

Ras homolog enriched in brain (RHEB) is a member of the RAS-family GTPases and was initially identified as an immediate-early gene expressed in the brain (17). RHEB functions as a positive regulator of the mechanistic target of rapamycin complex 1 (mTORC1) in mammals (18, 19, 20, 21). Although the regulatory mechanism of GTP loading onto RHEB remains to be determined, the GTP-bound form of RHEB activates mTORC1 on lysosomal membranes upon stimulation by insulin or growth factors (22, 23). The activated mTORC1 phosphorylates its substrates, such as the ribosomal protein S6 kinase (S6K), promoting protein synthesis and cell proliferation. RHEB activity is negatively regulated by the TSC complex, which acts as a GAP for RHEB (20, 24, 25). In the absence of insulin or growth factors, the TSC complex stimulates RHEB GTPase activity, leading to the conversion of the active RHEB-GTP to the inactive RHEB-GDP. Two RHEB mutations (RHEB/P37L and RHEB/S68P) were recently reported as being involved in psychiatric disorders, such as autism (26, 27). The expression of RHEB/P37L or RHEB/S68P increased cell size, suggesting that both mutants are gain-of-function mutants. However, the effects of these mutations on the biochemical properties of RHEB remain to be clarified.

The present study reported an HPLC-based assay that is broadly applicable to the analysis of the guanine-nucleotide bound status of small GTPases. Using this assay, we investigated the disease-associated RHEB/P37L and RHEB/S68P mutations, demonstrating that these mutations lead to RHEB activation in a distinct manner.

## Results

### Quantification of GDP and GTP using ion-pair reversed-phase high-performance liquid chromatography

**Figure 1:**
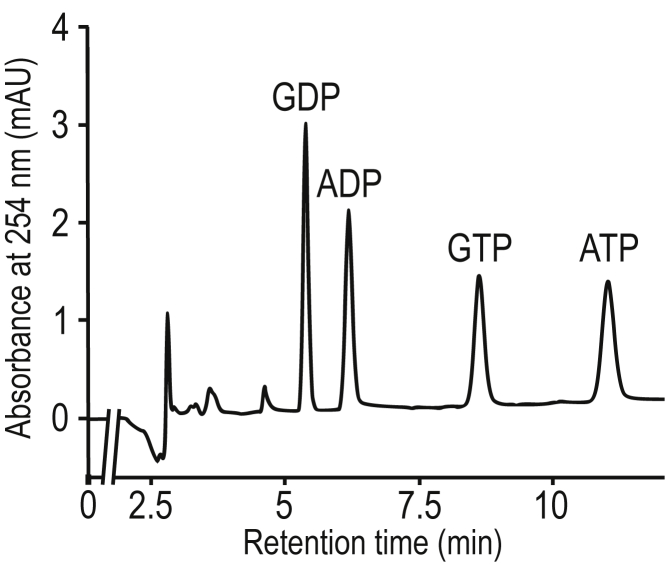
**Separation of adenine and guanine nucleotides by IP-RP-HPLC.** Representative chromatogram of a standard mixture of four nucleotides (ADP, ATP, GDP, and GTP, each 10 pmol) using IP-RP-HPLC. The nucleotides were detected by a UV detector at a wavelength of 254 nm. The peaks of GDP, ADP, GTP, and ATP were detected at 5.4, 6.2, 8.6, and 10.9 min, respectively, on chromatograms. IP-RP-HPLC, ion-pair reversed-phase HPLC.

We sought to analyze the activation state of small GTPases by directly quantifying the GDP and GTP bound to them. For this purpose, we performed ion-pair reversed-phase HPLC (IP-RP-HPLC) to measure GDP and GTP. Ion-pair reagents bind with counter-ions of charged compounds (such as nucleotides) to neutralize their charge, which allows the retention of the nucleotides in the reverse-phase columns, for analysis (28, 29). Under our optimized IP-RP-HPLC condition, a mixture of four nucleotides (GDP, GTP, ADP, and ATP; 10 pmol each) was well separated (Figs. 1 and S1). We determined the retention time, CV, and limit of quantification of all standard substances (Table 1). The correlation coefficient (*r*^2^) obtained from the linear regression of the calibration concentration range was ≥0.999 for GDP and GTP (0.5–100 pmol).

**Table 1:** Analytical data for the four nucleotides standards using IP-RP-HPLC

Nucleotide	Retention time	CV^a^	LoQ^b^
GDP	5.4 min	1.21%	0.47 pmol
GTP	8.6 min	1.21%	0.50 pmol
ADP	6.2 min	1.40%	0.27 pmol
ATP	10.9 min	2.64%	0.34 pmol

a Coefficient of variation.

b Limit of quantification.

### Analysis of the guanine-nucleotide-bound state of RAS-family GTPases in cells

**Figure 2:**
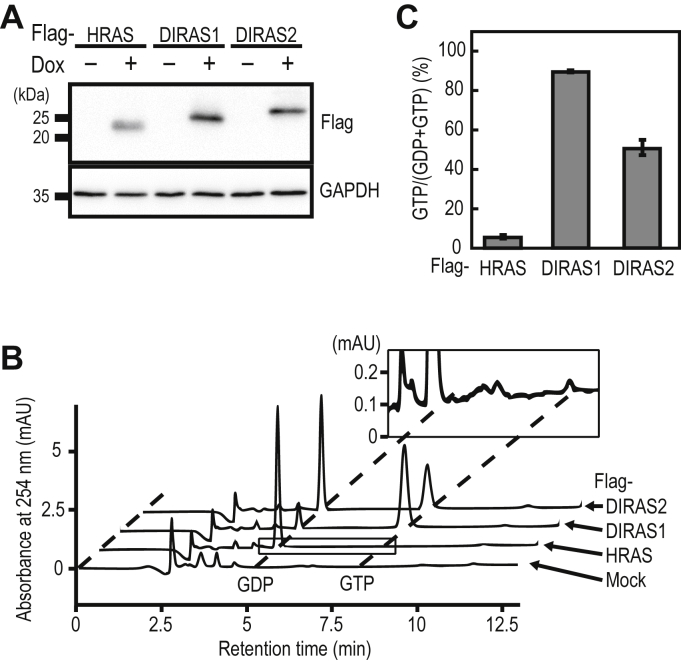
**Analysis of the guanine-nucleotide-bound forms of RAS-family GTPases in HeLa cells.***A*, Dox-dependent expression of Flag-tagged HRAS, DIRAS1, and DIRAS2 in HeLa cells. The cell lines expressing the indicated proteins in a Dox-dependent manner were cultured in the absence or presence of 1 μg/ml of Dox for 24 h, and the cell lysates were subjected to Western blot analysis using anti-Flag and anti-GAPDH antibodies. *B*, representative chromatogram of guanine nucleotides bound to Flag-tagged HRAS, DIRAS1, and DIRAS2 in HeLa cells. Anti-Flag immunoprecipitates from the indicated cell lines were subjected to IP-RP-HPLC analysis. To visualize the GTP signal of HRAS, a magnified view of the enclosed area of HRAS is shown as the *inset*. *C*, the relative amounts of guanine nucleotides associated with Flag-tagged proteins were quantified from the peak areas of GDP and GTP using IP-RP-HPLC. The data are the means ± SD from three independent experiments. IP-RP-HPLC, ion-pair reversed-phase HPLC; Dox, doxycycline.

We tested whether the guanine nucleotides bound to small GTPases can be quantified using IP-RP-HPLC. Our previous study using metabolic labeling with [^32^P]orthophosphate has shown that HRAS existed predominantly in the GDP-bound form (30). In contrast, DIRAS1 and DIRAS2 displayed considerable GTP-bound forms, probably because of the accelerated intrinsic guanine-nucleotide exchange reaction. Thus, we thought that these small GTPases with different ratios of GTP-bound forms would be suitable for validating IP-RP-HPLC analysis. We established HeLa cell lines expressing Flag-tagged RAS-family small GTPases (HRAS, DIRAS1, and DIRAS2) in a doxycycline (dox)-dependent manner (Fig. 2*A*). The Flag-tagged proteins were immunopurified from the corresponding cell lysates and denatured by heat to dissociate the bound guanine nucleotides. We could reproducibly quantify the amount of GDP and GTP bound to each small GTPase using IP-RP-HPLC (Fig. 2*B*). The ratios of the GTP-bound form of HRAS, DIRAS1, and DIRAS2 were 4.7%, 88.9%, and 50.2%, respectively (Fig. 2*C*), which were comparable with those obtained using metabolic labeling with [^32^P]orthophosphate (30). These results showed that IP-RP-HPLC can be used to analyze the guanine-nucleotide-bound status of small GTPases in cells.

### Analysis of the RHEB activation state in cells

**Figure 3:**
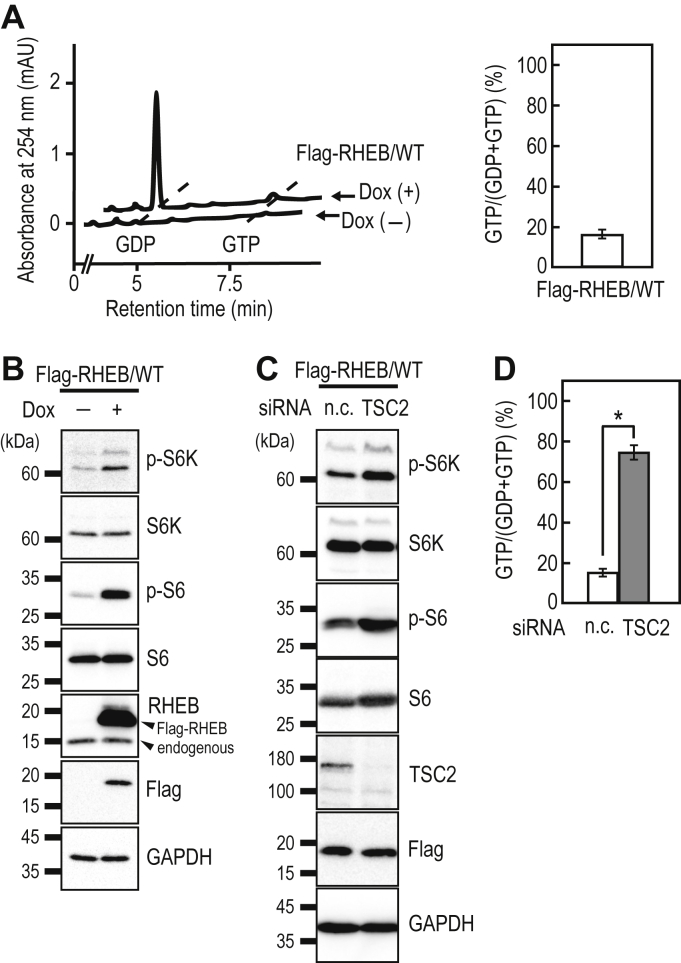
**Analysis of the activation status of Flag-RHEB in HeLa cells.***A*, cell lines expressing Flag-RHEB/WT in a Dox-dependent manner were cultured without or with 1 μg/ml of Dox for 24 h, and anti-Flag immunoprecipitates from the cell lysates were subjected to IP-RP-HPLC analysis. Representative chromatogram of guanine nucleotides bound to Flag-RHEB/WT in HeLa cells (*left panel*). The relative amounts of guanine nucleotides associated with Flag-RHEB/WT were quantified from the peak areas of GDP and GTP (*right panel*). The data are the means ± SD from three independent experiments. *B*, cell lines expressing Flag-RHEB/WT in a Dox-dependent manner were cultured without or with 1 μg/ml of Dox for 24 h, and the cell lysates were subjected to Western blot analysis using the indicated antibodies. *C* and *D*, effect of TSC2 knockdown on mTORC1 signaling and the guanine-nucleotide bound state of Flag-RHEB/WT. The cell lines expressing Flag-RHEB/WT in a Dox-dependent manner were transfected with negative control (n.c.) or TSC2 siRNAs and cultured for 48 h, followed by 24 h of culture in the presence of 1 μg/ml of Dox. The cell lysates were subjected to Western blot analysis (*C*) or IP-RP-HPLC analysis (*D*). The data are the means ± SD from three independent experiments. ∗ *p* < 0.05, Student’s *t* test. Dox, doxycycline; IP-RP-HPLC, ion-pair reversed-phase HPLC; mTORC1, mechanistic target of rapamycin complex 1; RHEB, Ras homolog enriched in brain.

The small GTPase RHEB functions as a molecular switch to regulate mTORC1 activity in mammals. The GTP-bound form of RHEB stimulates mTORC1 activity on lysosomal membranes (22, 23). In turn, the activated mTORC1 phosphorylates its substrates, such as S6K and the eukaryotic translation initiation factor 4E-binding protein 1, thus promoting protein synthesis and cell growth. RHEB inactivation is mediated by the TSC complex (consisting of TSC1, TSC2, and TBC1D7 in mammals), which functions as a GAP for RHEB *via* the TSC2 GAP domain (20, 25). To elucidate the guanine-nucleotide-bound state of RHEB in cells, we established a HeLa cell line expressing Flag-RHEB in a dox-dependent manner. We performed an IP-RP-HPLC analysis of Flag-RHEB proteins expressed in HeLa cells, which showed that the ratio of the GTP-bound form of Flag-RHEB was about 16% (Fig. 3*A*), which was comparable with those reported in previous studies (31). Dox-induced expression of Flag-RHEB enhanced the phosphorylation levels of S6K and S6 *via* mTORC1 activation (Fig. 3*B*), as reported previously (31). We further analyzed the effect of the loss of TSC2 on the guanine-nucleotide-bound state of Flag-RHEB. Previous studies have shown that loss of TSC2 causes RHEB activation *via* the suppression of RHEB-GTPase activity (20). In fact, RNAi against TSC2 (siTSC2) increased the proportion of the GTP-bound form of RHEB with a concomitant elevation of the phosphorylation levels of S6K and S6 (Fig. 3, *C* and *D*). These results indicate that IP-RP-HPLC can be used to analyze the RHEB activation status in cells.

### Disease-associated mutations in RHEB cause an increase in the GTP-bound form of RHEB

**Figure 4:**
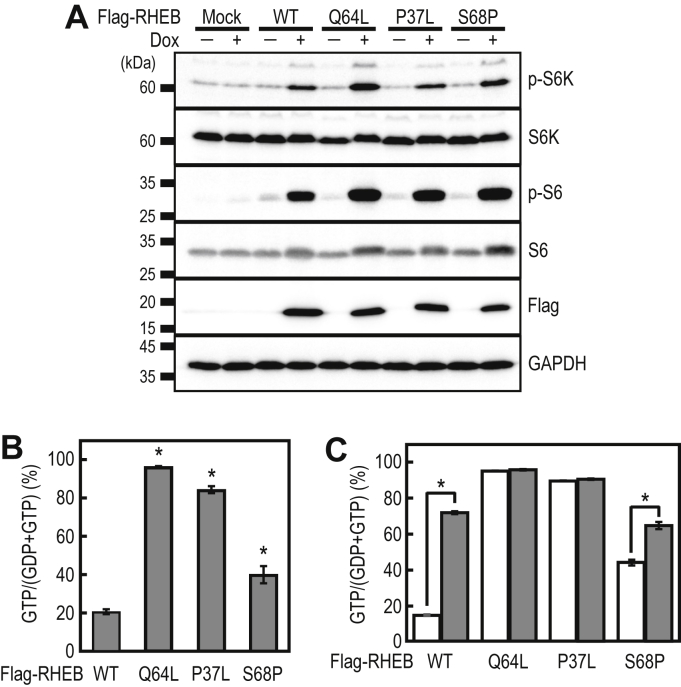
**Analysis of the activation status of the disease-associated RHEB mutants in HeLa cells.***A*, the cell lines expressing Flag-RHEB/WT or mutants in a Dox-dependent manner were cultured for 24 h in the absence or presence of 1 μg/ml of Dox, and the cell lysates were subjected to Western blot analysis using the indicated antibodies. *B*, anti-Flag immunoprecipitates from the cell lines expressing Flag-RHEB/WT and mutants were subjected to IP-RP-HPLC analysis. The data are the means ± SD from three independent experiments. ∗ *p* < 0.05, compared with WT by Dunnett’s multiple comparison test. *C*, cell lines expressing Flag-RHEB/WT and mutants in a Dox-dependent manner were transfected with negative control (*open bars*) or TSC2 (*closed bars*) siRNAs and cultured for 48 h, after 24 h of culture in the presence of 1 μg/ml of Dox. The cell lysates were subjected to anti-Flag immunoprecipitation for IP-RP-HPLC analysis. The data are the means ± SD from three independent experiments. ∗ *p* < 0.05, Student’s *t* test. IP-RP-HPLC, ion-pair reversed-phase HPLC; RHEB, Ras homolog enriched in brain.

A recent study reported that the P37L and S68P mutations in RHEB were associated with neurological disorders, such as autism (26). That research suggested that these mutations cause RHEB activation based on the enhanced mTORC1 activity detected in the RHEB-mutant-expressing cells. However, no detailed biochemical analysis has been performed using these RHEB mutants. Thus, we generated HeLa cell lines expressing Flag-tagged RHEB/P37L, RHEB/S68P, and RHEB/Q64L (an active RHEB mutant as a control) for their biochemical characterization. Ectopic expression of these mutants caused the activation of mTORC1 signaling (as estimated by the phosphorylation levels of S6K and S6), as reported previously (Fig. 4*A*) (20, 26, 27). The RHEB/Q64L mutant is less sensitive to the GAP activity of TSC2 than is RHEB/WT and exists predominantly in the GTP-bound form in cells (32). In fact, the IP-RP-HPLC analysis showed a marked increase in the ratio of the GTP-bound form of RHEB/Q64L compared with RHEB/WT (Fig. 4*B*). Furthermore, we found that both RHEB/P37L and RHEB/S68P showed increases in their GTP-bound forms (Fig. 4*B*). These results suggest that the P37L and S68P disease-associated mutations in RHEB cause its activation, thus leading to enhanced mTORC1 signaling.

### RHEB/P37L and RHEB/S68P are less sensitive to the TSC2-mediated GAP activity than is WT RHEB

To elucidate the mechanism underlying the increases in the ratio of the GTP-bound forms of the RHEB/P37L and RHEB/S68P mutants, we investigated the effects of the loss of TSC2 on the guanine-nucleotide-bound status of these mutants (Fig. 4*C*). Similar to RHEB/Q64L, the GTP-bound ratio of RHEB/P37L was unaffected by siTSC2, suggesting that the RHEB/P37L GTP hydrolysis is less sensitive to TSC2 GAP. In contrast, siTSC2 significantly increased the GTP-bound ratio of RHEB/S68P, suggesting that RHEB/S68P retained sensitivity to the GAP activity of TSC2 to some extent.

**Figure 5:**
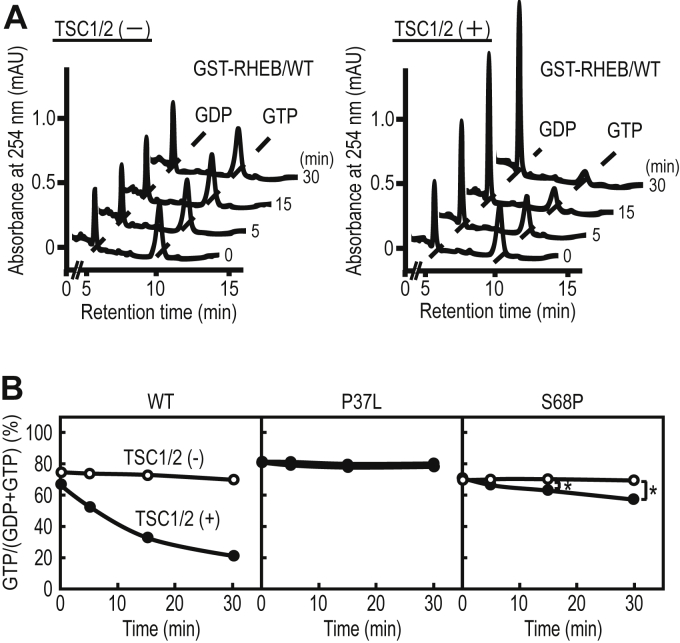
**Effect of the TSC1/2 complex on the GTPase activity of WT and mutant RHEB proteins.***A*, representative chromatograms of the GTPase assay using IP-RP-HPLC. The GST-fusion proteins of WT RHEB were incubated at 30 °C without (*left panel*) or with (*right panel*) Flag-TSC1/2 immunoprecipitation beads at the indicated times, and the aliquots were subjected to IP-RP-HPLC analysis. *B*, the GST-fusion proteins of the WT and mutant forms of RHEB were incubated at 30 °C without (*open circle*) or with (*closed circle*) Flag-TSC1/2 immunoprecipitation beads at the indicated times, and the aliquots were subjected to IP-RP-HPLC analysis. The data are the means from three independent experiments. The error bars are not shown as the standard deviations of the data were within 1%. ∗ *p* < 0.05, Student’s *t* test. IP-RP-HPLC, ion-pair reversed-phase HPLC; RHEB, Ras homolog enriched in brain.

To analyze the effect of TSC2 on the GTPase activity of RHEB mutants in greater detail, we performed an *in vitro* GAP assay using GST-RHEB and the Flag–TSC1/2 complex. To analyze RHEB-GTPase activity, GST-RHEB was incubated with or without Flag-TSC1/2, and the reaction mixture was subjected to IP-RP-HPLC analysis. The guanine-nucleotide bound form of GST-RHEB/WT showed no apparent changes in the absence of Flag-TSC1/2 (Fig. 5*A*, *left panel*), whereas the amounts of the GTP-bound form were time-dependently decreased with a concomitant increase in those of the GDP-bound form in the presence of Flag-TSC1/2 (Fig. 5*A*, *right panel*), indicating that Flag-TSC1/2 stimulated the GTPase activity of GST-RHEB/WT. We found that Flag-TSC1/2 did not affect the GTPase activity of GST-RHEB/P37L (Fig. 5*B*, *P37L*). In contrast, it weakly but significantly promoted that of GST-RHEB/S68P (Fig. 5*B*, *S68P*). We have obtained similar results using untagged RHEB proteins, indicating that the GST-tag does not interfere with the assay (Fig. S2). Together with the results of the RNAi experiments (Fig. 4*C*), these data indicate that the loss and reduction of the sensitivity of RHEB/P37L and RHEB/S68P to TSC2-GAP, respectively, are responsible for the increases in their GTP-bound forms.

### The RHEB/S68P mutation promotes the guanine-nucleotide exchange reaction

**Figure 6:**
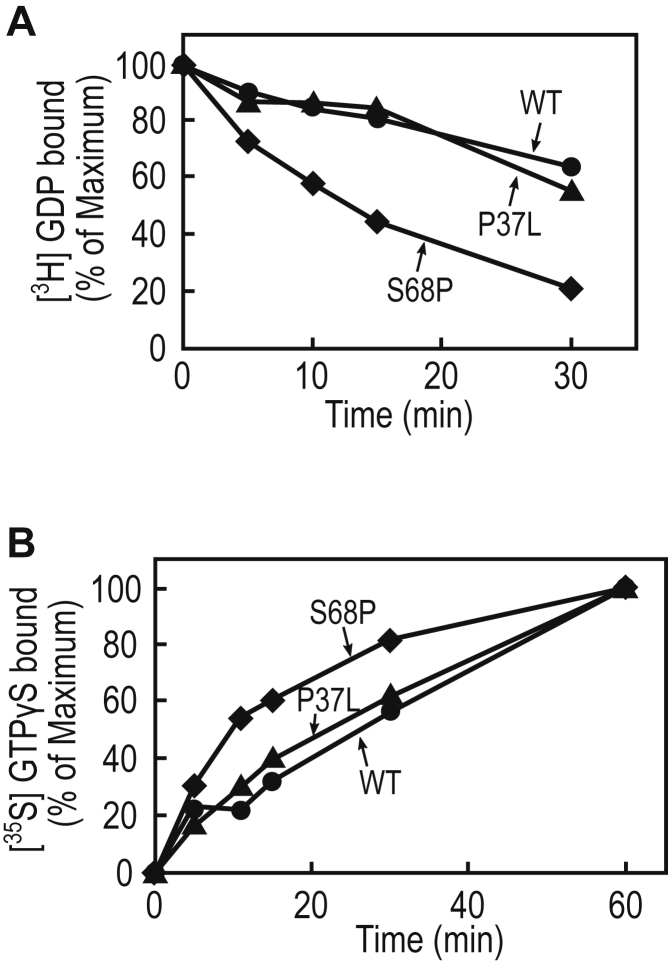
**Analysis of the GDP-dissociation and GTPγS-association rates of WT and mutant RHEB proteins.***A*, GDP-dissociation assay. The GST-fusion proteins of RHEB/WT (*circles*), P37L (*triangles*), or S68P (*rhombuses*) were incubated with 5 μM [^3^H]GDP in the presence of 5 mM Mg^2+^ for 20 min, and unlabeled GTPγS (200 μM) was added to the reaction mixture. At the indicated times, the aliquots (20 μl) were withdrawn and analyzed for [^3^H]GDP binding, as described in the “Experimental procedures.” The data are the means from two independent experiments. *B*, GTPγS-binding assay. The GST-fusion proteins of RHEB/WT (*circles*), P37L (*triangles*), or S68P (*rhombuses*) were incubated with 5 μM [^35^S]GTPγS for the indicated times, and [^35^S]GTPγS binding was determined, as described in the “Experimental procedures.” The data are the means from two independent experiments. RHEB, Ras homolog enriched in brain.

In addition to the decrease in GTP hydrolysis, the promotion of GDP/GTP exchange reactions can also cause an increase in the ratio of the GTP-bound forms of small GTPases. For example, HRAS/F28L exists mainly in a GTP-bound form because of an increased nucleotide-dissociation rate, despite its sensitivity to the GTPase activation proteins (33). Oncogenic RAC1 mutants, such as RAC1/P29S and RAC1/N92I, also display high GDP-dissociation rates, which leads to the existence of the mutants predominantly in their GTP-bound active states (10, 11, 12, 13). Thus, we investigated whether the acceleration of the GDP/GTP exchange rate is involved in the increases in the GTP-bound form of the RHEB/P37L and RHEB/S68P mutants. Therefore, we performed an *in vitro* GDP-dissociation assay using purified *Escherichia coli* recombinant RHEB proteins. We found that the dissociation rates of the WT and P37L proteins were comparable, whereas that of S68P was increased by comparison to them (Fig. 6*A*). Consistently, an *in vitro* [^35^S]GTPγS binding assay revealed that the GTPγS-binding rate of RHEB/S68P was enhanced compared with that of RHEB/WT and RHEB/P37L (Fig. 6*B*). These data indicate that the acceleration of the GDP/GTP exchange rate, together with a reduced sensitivity to TSC2-GAP, is involved in the increased ratio of the RHEB/S68P GTP-bound form in cells.

## Discussion

This study established a straightforward and versatile HPLC-based method to analyze the guanine-nucleotide binding state of small GTPases. The IP-RP-HPLC analysis enabled the quantification of picomolar levels of GDP and GTP bound to small GTPases. Using this method, we found that the disease-associated RHEB mutants (RHEB/P37L and RHEB/S68P) displayed an increased proportion of GTP-bound forms in cells. Furthermore, an *in vitro* analysis using purified proteins indicated that loss of sensitivity to TSC2 GAP for RHEB/P37L and both reduced sensitivity to TSC2 GAP and enhanced guanine-nucleotide exchange reaction for RHEB/S68P are involved in the increase of their GTP-bound forms, respectively. Thus, IP-RP-HPLC analysis will be an effective tool in the analysis of the activation status and regulatory mechanisms of various small GTPases.

Various methods have been developed to analyze the intracellular guanine-nucleotide binding states of small GTPases. The classic method consists in metabolic labeling using [^32^P]orthophosphate, in which small GTPases are loaded with radiolabeled GDP or GTP in cells (20, 30, 34). After immunoisolation of small GTPases from the cells, the bound GDP and GTP are separated by TLC to be analyzed by autoradiography. This method can detect GDP and GTP with high sensitivity; however, its drawbacks include the need for facilities to use radioisotopes, radiation effects on cells, and laborious and time-consuming procedures. Active small GTPases can be evaluated by GST pull-down assay using an effector domain fused to GST or conformation-specific antibodies that are capable of binding to active (GTP-bound) small GTPases (35, 36, 37, 38, 39, 40, 41). These assays can be performed conveniently without special facilities; however, they lack versatility because they require known effector proteins or specific antibodies. In addition, if a mutation of small GTPases alters the interaction with their probes (*i.e.*, GST-effector fusion proteins or conformation-specific antibodies), the results obtained with the assays will not reflect the guanine-nucleotide binding states of small GTPases. Precautions should also be taken regarding the specificity of the conformation-specific antibodies, because a lack of specificity can lead to a misleading interpretation of the obtained data (42). FRET is a powerful tool to analyze the activation state of small GTPases in a spatiotemporal manner (43, 44, 45, 46). However, it has limited versatility because it requires specific FRET probes for sensitive detection and cannot be used to evaluate endogenous small GTPases.

The IP-RP-HPLC analysis developed in this study is a versatile and quantitative method to measure the activation state of small GTPases without the need for special facilities or probes. This assay can be completed within 3 h (from immunoprecipitation to HPLC analysis), which is a relatively short time compared with that required by other methods. However, because the present study was performed *via* ectopic expression of Flag-tagged proteins, the physiological relevance of its results is limited. Tagging endogenous genes with genome-editing technology will be helpful to analyze the activation state of any small GTPases, in principle, at the endogenous expression level. Considering that the expression levels of endogenous small GTPases are relatively low, a highly sensitive detection system for GDP and GTP (*e.g.*, fluorescence derivatization) will also be needed.

We analyzed the intellectual-disability-related RHEB/P37L and RHEB/S68P mutants using IP-RP-HPLC. A previous study has shown that RHEB/P37L and RHEB/S68P cause an intellectual disability syndrome associated with megalencephaly (26). An *in vivo* analysis of these RHEB mutants using zebrafish and mice showed that expression of the mutants caused an increase in cell-body and head size, with concomitant hyperactivation of the mTOR pathway, indicating that RHEB/P37L and RHEB/S68P are gain-of-function mutants (26). Consistently, the present study revealed that RHEB/P37L and RHEB/S68P displayed an increased ratio of their GTP-bound forms in cells. An *in vitro* analysis using purified proteins also showed that both RHEB mutants were less sensitive to TSC2 GAP compared with WT RHEB. Previous studies have reported RHEB mutants (*e.g.*, RHEB/Q64L and RHEB/R15G) with low sensitivities to TSC2 GAP (32, 47). Their sensitivities to TSC2 GAP are reduced, but not eliminated. In contrast, RHEB/P37L was entirely resistant to TSC2 GAP. Consistently, a recent study showed that RHEB/P37L-induced S6K phosphorylation was not attenuated by TSC1/TSC2 overexpression (27). Substitution of Pro with Leu in KRAS (equivalent to P37L in RHEB) causes a loss of interaction between KRAS and RAS-GAP, which leads to the existence of KRAS/P34L primarily in a GTP-bound form in cells (48). Moreover, Hansmann *et al*. have reported that RHEB/Pro37 is vital for the interaction of the switch I region with the TSC2-GAP domain (49). Thus, the complete resistance to TSC2 observed for RHEB/P37L is likely because of the loss of interaction between the switch I region and the TSC2-GAP domain. Considering that the Pro in the switch I region (corresponding to RHEB/Pro37) is highly conserved among the RAS-subfamily GTPases, it may be a critical amino acid residue for the interaction of many RAS-subfamily GTPases with their GAPs.

Unlike RHEB/P37L, RHEB/S68P was partially resistant to TSC2 GAP. Mazhab-Jafari *et al*. have proposed that RHEB/Asp65 is a candidate catalytic residue for GTP hydrolysis (50). Those authors also found that the RHEB/Ser68 side chain is responsible for stabilizing the Asp65 conformations. Thus, the substitution of RHEB/Ser68 with Pro may alter the conformation of Asp65, which prevents Asp65 from contributing to GTP hydrolysis, leading to reduced sensitivity to TSC2 GAP. RHEB/S68P also showed an acceleration of the guanine-nucleotide exchange reaction, with an enhanced GDP-dissociation rate. In RAS GTPases, amino acid mutations that reduce the affinity for Mg^2+^ can enhance the guanine-nucleotide exchange reaction, leading to a higher proportion of GTP-bound forms in cells (33, 51, 52). Mg^2+^ is stabilized by the Asp side chain of the DxxG motif in the switch II region of HRAS, and substitution of the Asp with Ala perturbs the guanine-nucleotide binding (52). The RHEB/G63A mutation in the DxxG motif also accelerates the guanine-nucleotide exchange reactions (50). Considering that RHEB/Ser68 is close to the DxxG motif and that the substitution of RHEB/Ser68 with Pro can restrict the dihedral angle of the peptide main chain, the RHEB/S68P mutation may cause a conformational change in the switch II region, leading to the acceleration of the GDP-dissociation rate with the reduction of its affinity for Mg^2+^. In fact, we found that RHEB/S68P did not efficiently bind to GDP under a low Mg^2+^ concentration (data not shown). Further structural analysis by crystallography or NMR will be necessary to elucidate the details of the guanine-nucleotide-binding properties of the disease-associated RHEB mutants.

Reijnders *et al*. analyzed the phenotype of mice overexpressing RHEB/P37L or RHEB/S68P as a mouse model of megalencephaly (26). They found that the P37L mutant had a more severe phenotype with a higher frequency of seizures than did the S68P mutant. Our findings that RHEB/P37L exhibited a higher ratio of GTP-bound forms in cells compared with RHEB/S68P were correlated with the expressivity of the phenotype in mice expressing RHEB mutants. Recently, compounds that bind to RHEB and suppress mTORC1 signaling have been reported (53). IP-RP-HPLC analysis will help evaluate such compounds regarding their effects on the guanine-nucleotide-bound state of RHEB in cells. Although small GTPases have been conceived to be “undruggable” for a long time, compounds targeting oncogenic KRAS 4B/G12C have recently been identified and are undergoing preclinical and clinical trials (54, 55, 56). For drug discovery targeting small GTPases, it will be necessary to precisely evaluate their activation states in cells. The IP-RP-HPLC analysis presented here may provide an effective means to that end in terms of versatility and quantification.

## Experimental procedures

### Ion-pair reverse-phase HPLC analysis conditions

The IP-RP-HPLC system comprised a DGU-20A3 degasser, an LC-20AD pump, a SIL-20AC autosampler, a CTO-20AC column oven, an SPD-20A UV/VIS detector, and a CBM-20A Communication Bus Module (Shimadzu). Instrumental control and data analysis were performed using LC Solution (Shimadzu). The nucleotides were separated using a Gemini 5 μm NX-C18 110 Å LC Column (150 × 4.6 mm) (Phenomenex, 00F-4454-E0) with a Gemini NX C18 Security Guard Cartridge (4.0 × 3.0 mm) (Phenomenex, AJ0-8368). The isocratic mobile phase consisted of 20% acetonitrile and 80% 30 mM phosphate buffer (pH 7.0) containing 10 mM tetrabutylammonium bromide (TBA-B). Isocratic elution was performed at 40 °C with a flow rate of 0.8 ml/min.

### Plasmids, siRNAs, and antibodies

Plasmids to establish stable cell lines were constructed using the Gateway cloning technology (Thermo Fisher Scientific). pENTR-TiTRE and pENTR-TRE3G-BI were used as donor plasmids for tightly controlled gene expression by a tetracycline-responsive promoter and simultaneous expression of two genes by a bi-directional tetracycline-responsive promoter, respectively. The donor plasmids for the expression of Flag-tagged HRAS, DIRAS1, DIRAS2, RHEB, and RHEB mutants were constructed by cloning each gene into the *Eco*RI/*Sal*I site of pENTR-TiTRE. A donor plasmid for the simultaneous expression of TSC1-Myc and Flag-TSC2 was constructed by cloning TSC1-Myc and Flag-TSC2 into the *Eco*RI*/Sal*I and *Pac*I sites of pENTR_TRE3G-BI, respectively. The DNA fragments of TSC1-Myc and Flag-TSC2 were amplified by PCR using pcDNA3.1 myc TSC1 (a gift from Cheryl Walker, Addgene plasmid #12133; http://n2t.net/addgene:12133; RRID:Addgene_12133) and pcDNA3 Flag-TSC2 (a gift from Brendan Manning, Addgene plasmid # 14129; http://n2t.net/addgene:14129; RRID:Addgene_14129) as a template, respectively (57, 58). The destination plasmid pAAVS1_Puro_ccdb_Ubc_rtTA was constructed as follows: the attR1-ccdb-attR2-Ubc-rtTA cassette from pSLIK-Neo (ATCC, MBA-235) was cloned into the *Nsi*I*/Pme*I site of AAVS1_Puro_PGK1_3xFLAG_Twin_Strep (a gift from Yannick Doyon, Addgene plasmid #68375; http://n2t.net/addgene:68375; RRID:Addgene_68375) (59). Destination plasmids for expression in HeLa cells or HEK293T cells were obtained by LR clonase (Thermo Fisher Scientific) reaction of each donor plasmid with the destination plasmid pAAVS1_Puro_ccdb_Ubc_rtTA, according to the manufacturer’s protocol. Plasmids for the expression of the RHEB recombinant protein in *E. coli* were constructed by cloning the DNA fragments corresponding to amino acids 1 to 180 of RHEB into the pCold GST vector (Takara Bio). The pCas Guide AAVS1-T2 plasmid was constructed by cloning AAVS1-T2 gRNA fragment into the pCas-Guide vector (OriGene).

The following antibodies were used: anti-RHEB (Cell Signaling Technology, #13879), anti-S6K (Cell Signaling Technology, #2708), anti-phospho S6K (Thr389) (Cell Signaling Technology, #9234), anti-S6 (Cell Signaling Technology, #2217), anti-phospho S6 (Ser235/Ser236) (Cell Signaling Technology, #4858), anti-TSC2 (Tuberin) (Santa Cruz, sc-893), anti-DYKDDDDK (Flag) (Wako, clone 1E6), and anti-GAPDH (Wako, clone 5A12). Horseradish peroxidase-conjugated secondary antibodies were purchased from The Jackson Laboratory.

### Western blotting

The cell lysates were separated on SDS–polyacrylamide gels and transferred to ClearTrans SP PVDF Membranes (Wako) using a Trans-Blot Turbo Transfer System (BIORAD). After blocking with 3% skim milk or 5% bovine serum albumin (BSA) (for anti-phospho S6K and anti-phospho S6) in TBS (20 mM Tris-HCl (pH7.5) and 150 mM NaCl) containing 0.1% Tween 20, the membranes were probed with antibodies and subjected to chemiluminescent measurement using EzWest LumiOne (ATTO) and LuminoGraph II (ATTO).

### Cell culture and transfection

HeLa and HEK293T cells were cultured in Dulbecco’s Modified Eagle’s Medium (DMEM) containing 10% fetal bovine serum, penicillin, and streptomycin. Isogenic stable cell lines expressing the gene of interest were generated by CRISPR/Cas9-driven targeted integration of the gene into the safe-harbor genomic locus *AAVS1*, as described previously (59). Briefly, HeLa or HEK293T cells were cotransfected with a destination plasmid for expression and a pCas Guide AAVS1-T2 plasmid (for expression of the Cas9 protein and AAVS1-T2 gRNA), and the cells with integrated DNA were selected using a culture medium containing puromycin (0.75 μg/ml).

### Immunoprecipitation of Flag-tagged small GTPases for IP-RP-HPLC

Cells that had been cultured in 100-mm dishes with 1 μg/ml of Dox for 24 h were lysed with 550 μl of ice-cold extraction buffer (40 mM Tris-HCl, pH 7.5, 100 mM NaCl, 5 mM MgCl_2_, 1% (w/v) Lubrol, 1 mM DTT, and 0.5 mM AEBSF). The cell lysates were centrifuged, and the supernatants were incubated with anti-DYKDDDDK tag antibody beads (10 μl bed volume, Wako) at 4 °C for 15 min. The beads were washed twice with ice-cold wash buffer 1 (40 mM Tris-HCl, pH 7.5, 100 mM NaCl, 5 mM MgCl_2_, 0.1% (w/v) Lubrol, and 1 mM DTT) and once with ice-cold wash buffer 2 (30 mM potassium phosphate buffer, pH 7.0, 100 mM NaCl, 1 mM MgCl_2_, and 0.1% (w/v) Lubrol). Flag-tagged small GTPases were eluted with 60 μl of an acidic solution (100 mM potassium phosphate buffer, pH 3.0, and 0.1% (w/v) Lubrol). The elution (50 μl) was neutralized with 11.2 μl of 0.5 M K_2_HPO_4_ and heat-denatured at 90 °C for 3 min, to release the guanine nucleotides from small GTPases. The denatured sample (46.2 μl) was mixed with 113.8 μl of distilled water and 1.6 μl of 1 M TBA-B, followed by filtration using an Amicon Ultra 30-kDa centrifugal filter (Merck Millipore). The flow-through fraction was then subjected to IP-RP-HPLC analysis.

### Expression and purification of GST-fusion proteins

GST-RHEB fusion proteins were prepared, as described previously (30), with slight modifications. A preculture of *E. coli* BL21-CodonPlus DE3 (Stratagene) cells containing the expression plasmid was diluted 1:50 with LB medium containing 50 μg/ml of ampicillin and grown at 37 °C for 2 h (A600 = ∼0.5), followed by induction with 0.1 mM IPTG at 16 °C for 16 h. The cultured cells (80 ml of culture) were collected by centrifugation and suspended in 10 ml of buffer A (50 mM Tris-HCl (pH 7.5), 100 mM NaCl, and 5 mM MgCl_2_) containing 0.5 mM AEBSF and sonicated for 5 min in ice-cold water. After centrifugation (200,000*g*, 30 min at 4 °C), the supernatant was applied to a glutathione-Sepharose 4B (Cytiva) column (∼0.4-ml bed) that had been equilibrated with buffer A. After washing the column with the same buffer, the proteins were eluted from the column with 1.6 ml of elution buffer (100 mM Tris-HCl (pH 8.0), 50 mM NaCl, 5 mM MgCl_2_, and 12.5 mM glutathione). After concentrating the eluted proteins using an Amicon Ultra 30-kDa centrifuge filter device (Millipore), the proteins were applied to a NAP-5 gel filtration column (Cytiva) that had been equilibrated with buffer A. The fractions containing GST-fusion proteins were collected and stocked at −80 °C until use.

### GTPase assay

The TSC1/2 protein complex was prepared, as described previously (60), with slight modifications. The cells expressing both Flag-TSC2 and TSC1-myc in a Dox-dependent manner were cultured in the presence of 1 μg/ml Dox for 24 h and then treated with Hanks’ Balanced Salt Solution containing 1 μg/ml of Dox for 30 min. The cells were lysed in lysis buffer (10 mM Tris-HCl (pH 7.5), 100 mM NaCl, 1% NP-40, 2 mM EDTA, 1 mM DTT, and 0.5 mM AEBSF), and the TSC1/TSC2 complex was immunoprecipitated with the anti-Flag antibody beads (Wako) at 4 °C for 60 min. The immune complexes were washed twice with ice-cold wash buffer (20 mM Tris-HCl (pH 7.5), 800 mM NaCl, 1% NP-40, 2 mM EDTA, and 1 mM DTT), after two additional washes with ice-cold GAP assay buffer (20 mM Tris-HCl (pH 7.5), 50 mM NaCl, and 5 mM MgCl_2_) and were then subjected to the GAP assay. For the GAP assay, the purified GST-RHEB proteins (5 μg) were incubated with or without TSC1/2 bound beads at 30 °C in a total volume of 100 μl of GAP assay buffer containing 0.2 mg/ml BSA. After incubation for the indicated times, 20 μl of the reaction mixture was withdrawn and incubated at 90 °C for 3 min. The denatured samples were mixed with 100 μl of 30 mM phosphate buffer (pH 7.0) and centrifuged at 15,000 rpm for 1 min at 4 °C. The supernatants (120 μl) were mixed with 1.2 μl of 1 M TBA-B, followed by filtration using an Amicon Ultra 30-kDa centrifugal filter. The flow-through fraction was subjected to IP-RP-HPLC analysis.

### GDP-dissociation and GTPγS-binding assay

The [^3^H]GDP-dissociation assay was performed, as described previously (10), with slight modifications. The purified GST-fusion proteins (0.25 μM) were incubated at 30 °C with 5 μM radiolabeled nucleotides (2000 dpm/pmol [^3^H]GDP) for 20 min in a preloading solution (50 mM Tris-HCl (pH 7.5), 100 mM NaCl, 5 mM MgCl_2_, 10 mM EDTA (for the WT and P37L mutant) or without EDTA (for the S68P mutant), and 0.2 mg/ml BSA). [^3^H]GDP dissociation from the proteins was initiated by the addition of unlabeled GTPγS to a final concentration of 200 μM. After incubation for the indicated times, 40 μl of the reaction mixture was withdrawn and diluted with 800 μl of ice-cold TMN buffer (20 mM Tris-HCl (pH 7.5), 20 mM MgCl_2_, and 100 mM NaCl). The diluted sample was filtered through a membrane (0.45 μm pore size, mixed cellulose esters membrane, Millipore). The membrane was washed four times with 2 ml of the ice-cold TMN buffer and dried at 68 °C. The radioactivity retained on the membrane was determined by a liquid scintillation counter Hidex600SL (HIDEX).

The GTPγS-binding assay was performed, as described previously (10), with slight modifications. The purified GST-fusion proteins (0.25 μM) were incubated at 30 °C with 5 μM radiolabeled nucleotides (5550 dpm/pmol [^35^S]GTPγS) in a 40 μl reaction mixture (50 mM Tris-HCl (pH 7.5), 100 mM NaCl, 5 mM MgCl_2_, and 0.2 mg/ml BSA). After incubation for the indicated times, the samples were diluted with 800 μl ice-cold TMN buffer and subjected to filtration and radioactivity measurement, as described in the GDP-dissociation assay.

## Data availability

Representative experiments are contained within the article. For any additional information, please contact the corresponding author.

## Supporting information

This article contains supporting information.

## Conflict of interest

The authors declare that they have no conflicts of interest with the contents of this article.

## References

[1] Colicelli J. (2004). Human RAS superfamily proteins and related GTPases. Sci. STKE.

[2] Wennerberg K., Rossman K.L., Der C.J. (2005). The Ras superfamily at a glance. J. Cell Sci..

[3] Cherfils J., Zeghouf M. (2013). Regulation of small GTPases by GEFs, GAPs, and GDIs. Physiol. Rev..

[4] Boulter E., Garcia-Mata R., Guilluy C., Dubash A., Rossi G., Brennwald P.J., Burridge K. (2010). Regulation of Rho GTPase crosstalk, degradation and activity by RhoGDI1. Nat. Cell Biol..

[5] Müller M.P., Goody R.S. (2017). Molecular control of Rab activity by GEFs, GAPs and GDI. Small GTPases.

[6] Sztul E., Chen P.-W., Casanova J.E., Cherfils J., Dacks J.B., Lambright D.G., Lee F.-J.S., Randazzo P.A., Santy L.C., Schürmann A., Wilhelmi I., Yohe M.E., Kahn R.A. (2019). ARF GTPases and their GEFs and GAPs: Concepts and challenges. Mol. Biol. Cell.

[7] Hobbs G.A., Der C.J., Rossman K.L. (2016). RAS isoforms and mutations in cancer at a glance. J. Cell Sci..

[8] Krauthammer M., Kong Y., Ha B.H., Evans P., Bacchiocchi A., McCusker J.P., Cheng E., Davis M.J., Goh G., Choi M., Ariyan S., Narayan D., Dutton-Regester K., Capatana A., Holman E.C. (2012). Exome sequencing identifies recurrent somatic RAC1 mutations in melanoma. Nat. Genet..

[9] Hodis E., Watson I.R., Kryukov G.V., Arold S.T., Imielinski M., Theurillat J.-P., Nickerson E., Auclair D., Li L., Place C., DiCara D., Ramos A.H., Lawrence M.S., Cibulskis K., Sivachenko A. (2012). A landscape of driver mutations in melanoma. Cell.

[10] Kawazu M., Ueno T., Kontani K., Ogita Y., Ando M., Fukumura K., Yamato A., Soda M., Takeuchi K., Miki Y. (2013). Transforming mutations of RAC guanosine triphosphatases in human cancers. Proc. Natl. Acad. Sci. U. S. A..

[11] Davis M.J., Ha B.H., Holman E.C., Halaban R., Schlessinger J., Boggon T.J. (2013). RAC1P29S is a spontaneously activating cancer-associated GTPase. Proc. Natl. Acad. Sci. U. S. A..

[12] Toyama Y., Kontani K., Katada T., Shimada I. (2019). Conformational landscape alternations promote oncogenic activities of Ras-related C3 botulinum toxin substrate 1 as revealed by NMR. Sci. Adv..

[13] Toyama Y., Kontani K., Katada T., Shimada I. (2019). Decreased conformational stability in the oncogenic N92I mutant of Ras-related C3 botulinum toxin substrate 1. Sci. Adv..

[14] Fan Y., Esmail M.A., Ansley S.J., Blacque O.E., Boroevich K., Ross A.J., Moore S.J., Badano J.L., May-Simera H., Compton D.S., Green J.S., Lewis R.A., Haelst M. M. van, Parfrey P.S., Baillie D.L. (2004). Mutations in a member of the Ras superfamily of small GTP-binding proteins causes Bardet-Biedl syndrome. Nat. Genet..

[15] Kobayashi T., Hori Y., Ueda N., Kajiho H., Muraoka S., Shima F., Kataoka T., Kontani K., Katada T. (2009). Biochemical characterization of missense mutations in the Arf/Arl-family small GTPase Arl6 causing Bardet–Biedl syndrome. Biochem. Biophys. Res. Commun..

[16] Wiens C., Tong Y., Esmail M., Oh E., Gerdes J.M., Wang J., Tempel W., Rattner J.B., Katsanis N., Park H.-W., Leroux M.R. (2010). Bardet-Biedl syndrome-associated small GTPase ARL6 (BBS3) functions at or near the ciliary gate and modulates Wnt signaling. J. Biol. Chem..

[17] Yamagata K., Sanders L.K., Kaufmann W.E., Yee W., Barnes C.A., Nathans D., Worley P.F. (1994). Rheb, a growth factor- and synaptic activity-regulated gene, encodes a novel Ras-related protein. J. Biol. Chem..

[18] Stocker H., Radimerski T., Schindelholz B., Wittwer F., Belawat P., Daram P., Breuer S., Thomas G., Hafen E. (2003). Rheb is an essential regulator of S6K in controlling cell growth in Drosophila. Nat. Cell Biol..

[19] Saucedo L.J., Gao X., Chiarelli D.A., Li L., Pan D., Edgar B.A. (2003). Rheb promotes cell growth as a component of the insulin/TOR signalling network. Nat. Cell Biol..

[20] Inoki K., Li Y., Xu T., Guan K.-L.L. (2003). Rheb GTPase is a direct target of TSC2 GAP activity and regulates mTOR signaling. Genes Dev..

[21] Long X., Lin Y., Ortiz-Vega S., Yonezawa K., Avruch J. (2005). Rheb binds and regulates the mTOR kinase. Curr. Biol..

[22] Menon S., Dibble C.C., Talbott G., Hoxhaj G., Valvezan A.J., Takahashi H., Cantley L.C., Manning B.D. (2014). Spatial control of the TSC complex integrates insulin and nutrient regulation of mTORC1 at the lysosome. Cell.

[23] Angarola B., Ferguson S.M. (2020). Coordination of Rheb lysosomal membrane interactions with mTORC1 activation. F1000Res..

[24] Thomas G., Hall M.N. (1997). TOR signalling and control of cell growth. Curr. Opin. Cell Biol..

[25] Dibble C.C., Elis W., Menon S., Qin W., Klekota J., Asara J.M., Finan P.M., Kwiatkowski D.J., Murphy L.O., Manning B.D. (2012). TBC1D7 is a third subunit of the TSC1-TSC2 complex upstream of mTORC1. Mol. Cell.

[26] Reijnders M., Kousi M., Woerden G. van, Klein M., Bralten J., Mancini G., Essen T. van, Proietti-Onori M., Smeets E., Gastel M. van, Stegmann A., Stevens S., Lelieveld S., Gilissen C., Pfundt R. (2017). Variation in a range of mTOR-related genes associates with intracranial volume and intellectual disability. Nat. Commun..

[27] Onori M.P., Koene L.M.C., Schäfer C.B., Nellist M., Velze M. de B. van, Gao Z., Elgersma Y., Woerden G. M. van (2021). RHEB/mTOR hyperactivity causes cortical malformations and epileptic seizures through increased axonal connectivity. PLoS Biol..

[28] Werner A. (1993). Reversed-phase and ion-pair separations of nucleotides, nucleosides and nucleobases: Analysis of biological samples in health and disease. J. Chromatogr..

[29] Contreras-Sanz A., Scott-Ward T.S., Gill H.S., Jacoby J.C., Birch R.E., Malone-Lee J., Taylor K.M., Peppiatt-Wildman C.M., Wildman S.S. (2012). Simultaneous quantification of 12 different nucleotides and nucleosides released from renal epithelium and in human urine samples using ion-pair reversed-phase HPLC. Purinergic Signal..

[30] Kontani K., Tada M., Ogawa T., Okai T., Saito K., Araki Y., Katada T. (2002). Di-Ras: A distinct subgroup of Ras-family GTPases with unique biochemical properties. J. Biol. Chem..

[31] Long X., Lin Y., Ortiz-Vega S., Busch S., Avruch J. (2007). The Rheb switch 2 segment is critical for signaling to target of rapamycin complex 1. J. Biol. Chem..

[32] Li Y., Inoki K., Guan K.-L.L. (2004). Biochemical and functional characterizations of small GTPase Rheb and TSC2 GAP activity. Mol. Cell Biol..

[33] Reinstein J., Schlichting I., Frech M., Goody R.S., Wittinghofer A. (1991). p21 with a phenylalanine 28—leucine mutation reacts normally with the GTPase activating protein GAP but nevertheless has transforming properties. J. Biol. Chem..

[34] Satoh T., Endo M., Nakamura S., Kaziro Y. (1988). Analysis of guanine nucleotide bound to ras protein in PC12 cells. FEBS Lett..

[35] Taylor S.J., Resnick R.J., Shalloway D. (2001). Nonradioactive determination of Ras-GTP levels using activated ras interaction assay. Methods Enzymol..

[36] Benard V., Bokoch G.M. (2002). Assay of Cdc42, Rac, and Rho GTPase activation by affinity methods. Methods Enzymol..

[37] Takatsu H., Yoshino K., Toda K., Nakayama K. (2002). GGA proteins associate with Golgi membranes through interaction between their GGAH domains and ADP-ribosylation factors. Biochem. J..

[38] Schweitzer J.K., D’Souza-Schorey C. (2002). Localization and activation of the ARF6 GTPase during cleavage furrow ingression and cytokinesis. J. Biol. Chem..

[39] Qi Y., Liang Z., Wang Z., Lu G., Li G. (2015). Rab GTPases, methods and protocols. Methods Mol. Biol..

[40] Dietz D.M., Sun H., Lobo M.K., Cahill M.E., Chadwick B., Gao V., Koo J.W., Mazei-Robison M.S., Dias C., Maze I., Damez-Werno D., Dietz K.C., Scobie K.N., Ferguson D., Christoffel D. (2012). Rac1 is essential in cocaine-induced structural plasticity of nucleus accumbens neurons. Nat. Neurosci..

[41] Kuipers D., Mehonic A., Kajita M., Peter L., Fujita Y., Duke T., Charras G., Gale J.E. (2014). Epithelial repair is a two-stage process driven first by dying cells and then by their neighbours. J. Cell Sci..

[42] Baker M.J., Cooke M., Kreider-Letterman G., Garcia-Mata R., Janmey P.A., Kazanietz M.G. (2020). Evaluation of active Rac1 levels in cancer cells: A case of misleading conclusions from immunofluorescence analysis. J. Biol. Chem..

[43] Mochizuki N., Yamashita S., Kurokawa K., Ohba Y., Nagai T., Miyawaki A., Matsuda M. (2001). Spatio-temporal images of growth-factor-induced activation of Ras and Rap1. Nature.

[44] Aoki K., Matsuda M. (2009). Visualization of small GTPase activity with fluorescence resonance energy transfer-based biosensors. Nat. Protoc..

[45] Kiyokawa E., Aoki K., Nakamura T., Matsuda M. (2011). Spatiotemporal regulation of small GTPases as revealed by probes based on the principle of Förster resonance energy transfer (FRET): Implications for signaling and pharmacology. Annu. Rev. Pharmacol..

[46] Kim J., Lee S., Jung K., Oh W.C., Kim N., Son S., Jo Y., Kwon H.-B., Heo W.D. (2019). Intensiometric biosensors visualize the activity of multiple small GTPases *in vivo*. Nat. Commun..

[47] Marshall C.B., Ho J., Buerger C., Plevin M.J., Li G.-Y.Y., Li Z., Ikura M., Stambolic V. (2009). Characterization of the intrinsic and TSC2-GAP-regulated GTPase activity of Rheb by real-time NMR. Sci. Signal..

[48] Gremer L., Merbitz-Zahradnik T., Dvorsky R., Cirstea I.C., Kratz C.P., Zenker M., Wittinghofer A., Ahmadian M.R. (2010). Germline KRAS mutations cause aberrant biochemical and physical properties leading to developmental disorders. Hum. Mutat..

[49] Hansmann P., Brückner A., Kiontke S., Berkenfeld B., Seebohm G., Brouillard P., Vikkula M., Jansen F.E., Nellist M., Oeckinghaus A., Kümmel D. (2020). Structure of the TSC2 GAP domain: Mechanistic insight into catalysis and pathogenic mutations. Structure.

[50] Mazhab-Jafari M.T., Marshall C.B., Ho J., Ishiyama N., Stambolic V., Ikura M. (2014). Structure-guided mutation of the conserved G3-box glycine in Rheb generates a constitutively activated regulator of mammalian target of rapamycin (mTOR). J. Biol. Chem..

[51] Hall A., Self A.J. (1986). The effect of Mg2+ on the guanine nucleotide exchange rate of p21N-ras. J. Biol. Chem..

[52] John J., Rensland H., Schlichting I., Vetter I., Borasio G.D., Goody R.S., Wittinghofer A. (1993). Kinetic and structural analysis of the Mg(2+)-binding site of the guanine nucleotide-binding protein p21H-ras. J. Biol. Chem..

[53] Mahoney S.J., Narayan S., Molz L., Berstler L.A., Kang S.A., Vlasuk G.P., Saiah E. (2018). A small molecule inhibitor of Rheb selectively targets mTORC1 signaling. Nat. Commun..

[54] Canon J., Rex K., Saiki A.Y., Mohr C., Cooke K., Bagal D., Gaida K., Holt T., Knutson C.G., Koppada N., Lanman B.A., Werner J., Rapaport A.S., Miguel T.S., Ortiz R. (2019). The clinical KRAS(G12C) inhibitor AMG 510 drives anti-tumour immunity. Nature.

[55] Hallin J., Engstrom L.D., Hargis L., Calinisan A., Aranda R., Briere D.M., Sudhakar N., Bowcut V., Baer B.R., Ballard J.A., Burkard M.R., Fell J.B., Fischer J.P., Vigers G.P., Xue Y. (2020). The KRASG12C inhibitor MRTX849 provides insight toward therapeutic susceptibility of KRAS-mutant cancers in mouse models and patients. Cancer Discov..

[56] Hong D.S., Fakih M.G., Strickler J.H., Desai J., Durm G.A., Shapiro G.I., Falchook G.S., Price T.J., Sacher A., Denlinger C.S., Bang Y.-J., Dy G.K., Krauss J.C., Kuboki Y., Kuo J.C. (2020). KRASG12C inhibition with sotorasib in advanced solid tumors. N. Engl. J. Med..

[57] Cai S.-L., Tee A.R., Short J.D., Bergeron J.M., Kim J., Shen J., Guo R., Johnson C.L., Kiguchi K., Walker C.L. (2006). Activity of TSC2 is inhibited by AKT-mediated phosphorylation and membrane partitioning. J. Cell Biol..

[58] Manning B.D., Tee A.R., Logsdon M.N., Blenis J., Cantley L.C. (2002). Identification of the tuberous sclerosis complex-2 tumor suppressor gene product tuberin as a target of the phosphoinositide 3-kinase/Akt pathway. Mol. Cell.

[59] Dalvai M., Loehr J., Jacquet K., Huard C.C., Roques C., Herst P., Côté J., Doyon Y. (2015). A scalable genome-editing-based approach for mapping multiprotein complexes in human cells. Cell Rep..

[60] Li Y., Inoki K., Vikis H., Guan K. (2006). Measurements of TSC2 GAP activity toward Rheb. Methods Enzymol..

