# Supplementary material for: Development of a versatile HPLC-based method to evaluate the activation status of small GTPases
Source: J Biol Chem. 2021 Nov 19;297(6):101428. doi: 10.1016/j.jbc.2021.101428 (PMC8668980; doi:10.1016/j.jbc.2021.101428)
Supplement: Figures S1 and S2 [file mmc1.pdf]

Supporting Information

Title:

Development of a versatile HPLC-based method to evaluate the activation status of small GTPases

Authors:

Makoto Araki, Kaho Yoshimoto, Meguri Ohta, Toshiaki Katada, Kenji Kontani

Included materials:

Figure S1-S2

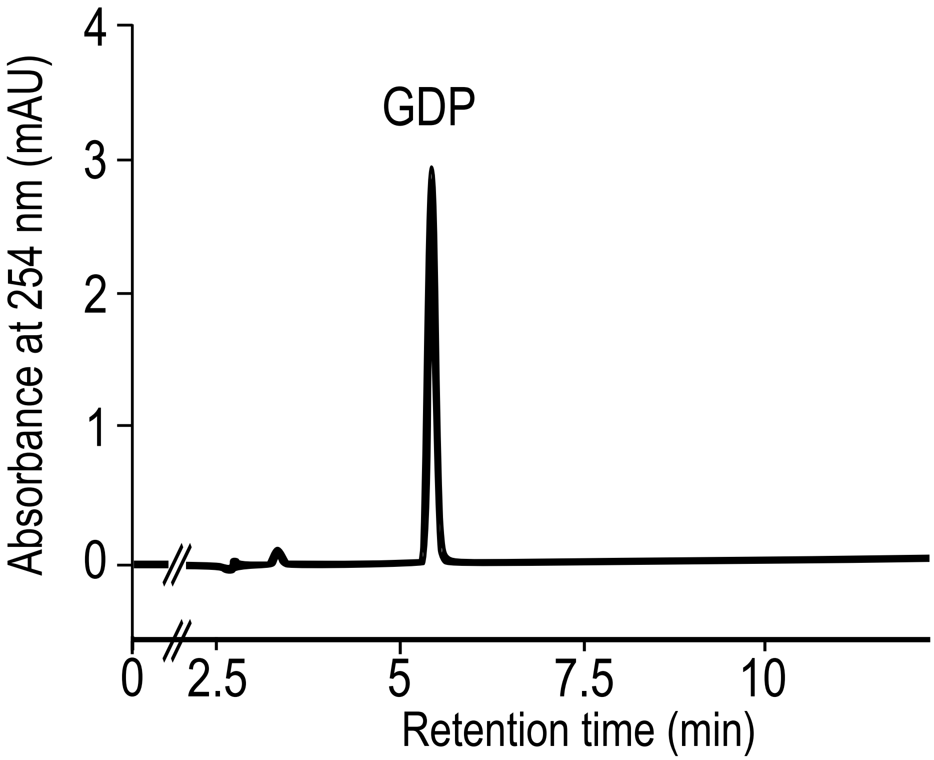

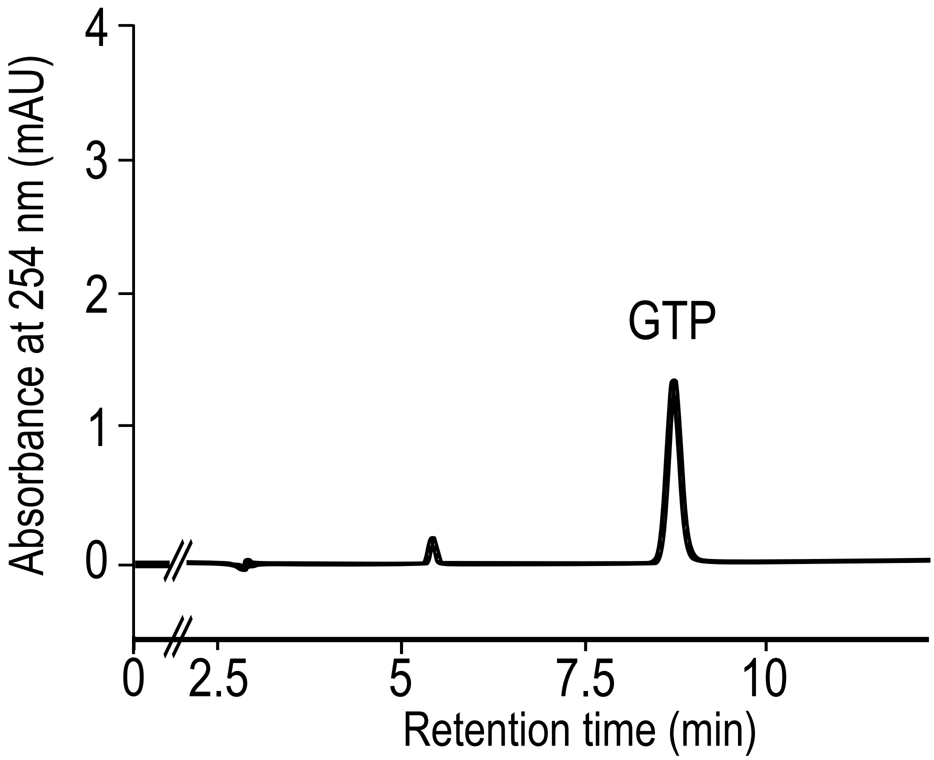

Figure S1. IP-RP-HPLC analysis of GDP and GTP.
Representative chromatograms of GDP (*upper panel*) and GTP (*lower panel*) (each 10 pmol) using IP-RP-HPLC. The nucleotides were detected by a UV detector at a wavelength of 254 nm. The peaks of GDP and GTP were detected at 5.4 and 8.6 min, respectively, on chromatograms.

Araki et al. Figure S1

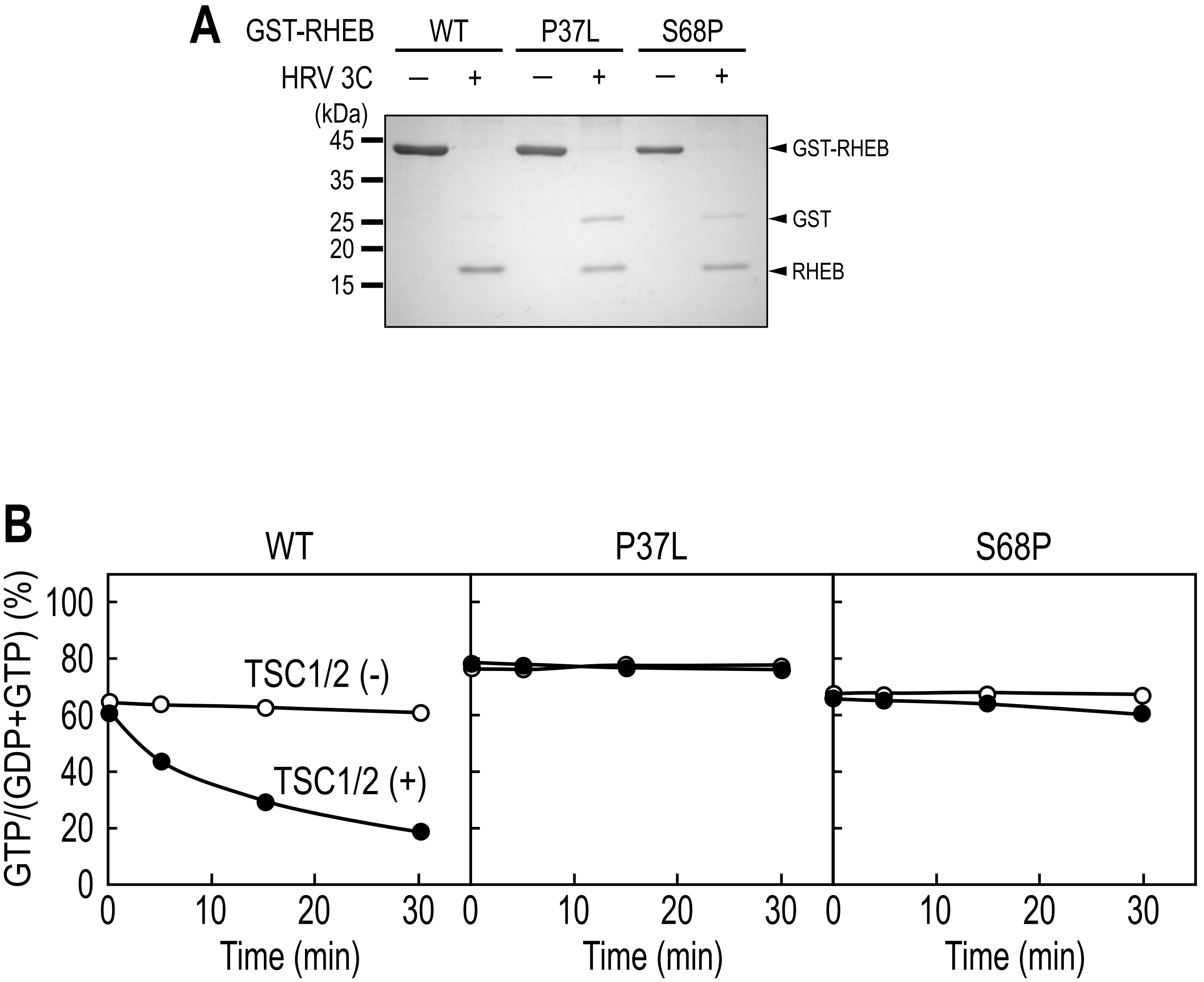

Figure S2. Preparation of untagged RHEB proteins and effect of the TSC1/2 complex on the RHEB GTPase activity.

(*A*) SDS-PAGE analysis of untagged RHEB proteins (Coomassie Brilliant Blue staining). GST-RHEB fusion proteins were completely cleaved with HRV3C protease, and the released GST was removed by incubation with Glutathione Sepharose 4B. Most of GST was removed from the mixture for RHEB/WT, whereas some amount of GST remained for RHEB/P37L and RHEB/S68P. (*B*) The untagged RHEB proteins were incubated at 30°C without (*open circle*) or with (*closed circle*) Flag-TSC1/2 immunoprecipitation beads at the indicated times, and the aliquots were subjected to IP-RP-HPLC analysis. Data are the means from two independent experiments.

Araki et al. Figure S2
